# Determination of the relative amounts of Gag and Pol proteins in foamy virus particles

**DOI:** 10.1186/1742-4690-2-44

**Published:** 2005-07-08

**Authors:** Marc Cartellieri, Wolfram Rudolph, Ottmar Herchenröder, Dirk Lindemann, Axel Rethwilm

**Affiliations:** 1 Institut für Virologie, Medizinische Fakultät, Technische, Universität Dresden, Germany; 2 Institut für Virologie und Immunbiologie, Universität Würzburg, Germany

## Abstract

We determined the relative ratios of Gag and Pol molecules in highly purified virions of spumaretroviruses or foamy viruses (FVs) using monoclonal antibodies and bacterially expressed reference proteins. We found that the cleaved p68^Gag ^moiety dominates in infectious FVs. Furthermore, approximate mean ratios in FV are 16:1 (pr71^Gag ^plus p68^Gag^:p85^RT^),12:1 (p68^Gag^:p85^RT^), and 10:1 (pr71^Gag ^plus p68^Gag^:p40^IN^). Thus, the results indicate that FVs have found a way to incorporate approximately as much Pol protein into their capsids as orthoretroviruses, despite a completely different Pol expression strategy.

## 

One of the central features of *Spumaretrovirinae*, which distinguishes them from *Orthoretrovirinae*, is the expression of a Pol precursor protein independently of the Gag protein from a spliced mRNA [1-3]. This mechanism of Pol generation raises several interesting questions: (i) How is Pol expression regulated? (ii) How is the Pol protein incorporated into the virion? (iii) And how much Pol protein is actually present in infectious viruses? While question one has, to our knowledge, not been investigated yet, answers to question two are emerging [4,5]. Here we tried to address question three.

Theoretical lines of argument favor the view that only a few molecules of Pol may be incorporated into a FV particle. The reverse transcriptase (RT) is the main enzymatic subunit of the Pol precursor [6]. This enzyme has been shown to be of much higher processivity than orthoretroviral RTs [7,8]. Therefore, it was argued that FVs probably encapsidate less of their highly active Pol protein compared to orthoretroviruses [7,8]. Following this line of argument, it is noteworthy that the FV protease (PR) is contained within the 85 kD Pol subunit, which also bears the RT/RNaseH [6]. However, in contrast to orthoretroviruses, the FV PR cleaves the cognate Gag protein only once prior to or during budding [6]. Therefore, FV may need less amounts of PR enzyme than orthoretroviruses.

Furthermore, experiments aimed to elucidate the mechanism of Pol protein particle incorporation (the above raised question two) indicated that Pol interacts with specific sequences on the (pre-) genomic RNA and that RNA serves as a bridging molecule between Gag (capsid) and Pol [4,5]. Two distinct elements on the RNA have been identified, which probably facilitate this interaction [4]. This can be regarded as another argument in support of only trace amounts of encapsidated Pol protein.

Here we wanted to investigate the approximate relative ratio of Pol to Gag molecules in infectious virions on a biochemical level to get an estimate of the FV particle composition using the prototypic FV (PFV) as a model. We did not attempt to determine absolute numbers of Gag and Pol molecules per particle.

### Prokaryotic expression and purification of viral proteins

The cloning strategy [9,10] and the purified recombinant proteins are depicted in Fig. 1. pETgag2 was made by digestion of pETgagl [11] with AdeI, T4 DNA polymerase treatment, and recutting with NdeI. A 1.9 kb *gag *gene (aa 1–625 of 648 aa) was inserted into pET22b (Novagen) in-frame to the C-terminal histidine tag after SacI, T4 DNA polymerase, and NdeI treatment. The PFV *pol *domain encoding the 85 kD PR, RT, and RNaseH subunits was amplified with primers #1217 (5'tc cacatatgaatcctcttcagctgttacagccgc) and #1414 (5'tattacactcgagcacataacttccttg), which bear NdeI and XhoI restriction sites (underlined). pETpol2 was made from pET22b and the amplimer using these enzymes. The integrase (IN; aa 751–1143) construct pETpol3 was made alike with #1219 (5'gttatgtgcatatgtgtaataccaaaaaacc) and #1413(5'tgcgctctcgagatttttttccaaatg). All plasmids were sequenced in their FV parts to verify correct insertions and to exclude PCR artifacts.

**Figure 1:**
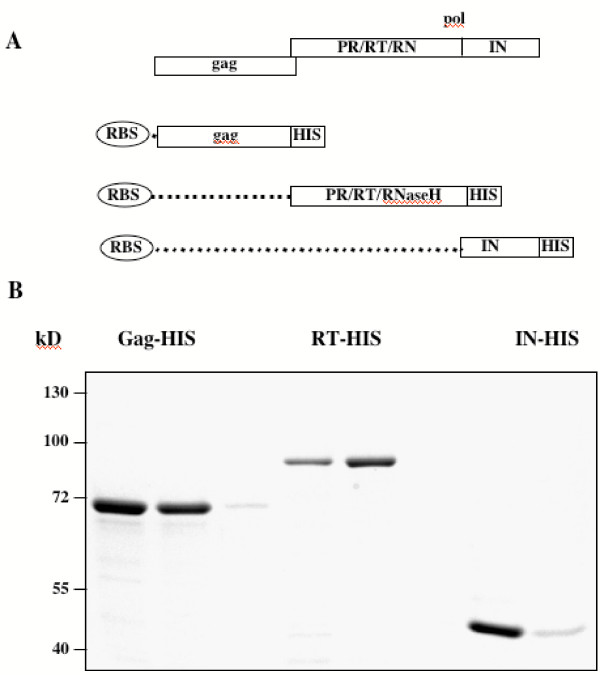
Bacterial expression of PFV *gag *and *pol *genes. (A) Strategy to insert the *gag *and *pol *open reading frames into the bacterial expression vector pET22b. The FV gene fragments are placed in frame to a C-terminal histidine (HIS) tag. (RBS), prokaryotic ribosomal binding site. (B) Coomassie stain of recombinant proteins which were purified via the C-terminal HIS-tag over Ni^2+^-chelate matrices. Two examples per protein are shown.

BL21(DE3)pLys (Novagen) served as a host strain for recombinant proteins. Expression was induced with 1 mM isopropyl-β-D-thiogalactopyranoside. The proteins were purified on Ni^2+^-chelate columns under denaturing conditions with 6 M urea. After renaturation in dialysis buffer (150 mM NaCl, 1 mM EDTA pH 5,0, 20 mM Tric-HCL pH 7,5) the amounts of purified proteins in the eluted fractions were determined by a BCA assay (Pierce). Proteins were subjected to sodium-dodecyl-sulfate-containing 7.5% polyacrylamide gel electrophoresis (SDS-PAGE) and Coomassie-blue stain. The purity was analyzed by digital imaging (Phoretix 1D Advanced Version 4.01).

### Pol protein is abundant in cells lytically infected with FV

We first estimated the amount of Pol proteins present in FV infected cells. In addition, we determined the sensitivity of the MABs in detecting Gag and Pol protein species. A cellular lysate was prepared from BHK-21 cells lytically infected with PFV, which was obtained by transfection of 293T cells with the pcHSRV2 infectious molecular clone by calcium phosphate coprecipitation [12]. Proteins in the lysates were analysed with the Gag and Pol hybridomas SGG1 (recognizing Gag), 15E10 (PR/RT/RnaseH), and 3E11 (IN) [11,13] in an immunoblot along with defined amounts of recombinant Gag and Pol proteins purified from bacteria. As shown in Fig. 2, the MAB 3E11 has a detection limit of approx. 10 ng of IN protein expressed in bacteria, while the RT (15E10) and Gag (SGG1) MABs were able to detect 20 ng and 40 ng of the respective proteins from bacteria. This experiment further revealed that the method to detect FV Gag and Pol by the ECLplus reagent (Amersham-Pharmacia) was in a linear range from 10 to more than 100 ng of recombinant protein (Fig. 2 and data not shown). The IgG concentrations of the hybridomas used in this particular experiment were determined, following a published protocol (Mouse-IgG-ELISA, Roche), to be 3.2μg/ml (3E11), 10.5 μg/ml (15E10), and 10.1 μg/ml (SGG1). In conclusion, the IN MAB was at least 12 times more sensitive than the Gag MAB and approx. 6.5 times more than the RT antibody.

**Figure 2:**
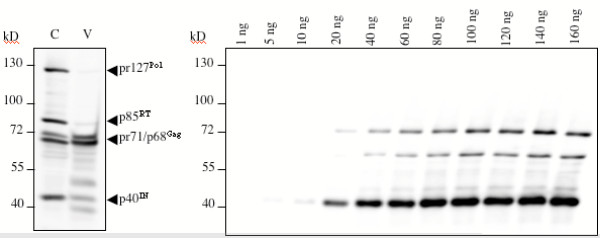
Immunoblot of a dilution series of recombinant Gag and Pol proteins, a cellular lysate (C), and extra-cellular virus (V) detected with the MABs SGG1 (Gag), 15E10 (RT), and 3E11 (IN). (C) was obtained by harvesting lytically infected BHK-21 cells, and (V) prepared by concentrating the supernatant of lytically infected cells through a sucrose cushion. On the right side the indicated amounts of recombinant proteins, specifying FV Gag and Pol proteins as shown in Fig. 1, were mixed and loaded onto an SDS-PAGE.

Due to the presence of five Gag and Pol molecule species of different molecular weights (pr71^Gag^, p68^Gag^, pr127^Po1^, p85^RT^, and p40^IN^) it was not possible to calculate exactly the respective molecule numbers present in infected cells. However, the comparison of the intensity of the lanes corresponding to Gag (pr71/p68) and Pol (pr127/p85/p40) proteins, which were detected by the MABs in the lysates, indicated that high amounts of Pol are expressed upon lytic infection in BHK-21 cells. This correlates well with the published amount of *pol*-specific mRNA, reported to equal the full-length or *gag*-specific mRNA in the bovine FV system [14]. The ease, with which Pol proteins can be detected in FV infected cells is indicative of their relatively high expression level compared to Gag. This finding questions the theoretical assumption of only trace amounts of Pol in FV particles. Obviously, FV utilizes distinct ways to avoid overloading infected cells with Pol protein. High cellular loads of retroviral Pol proteins can be associated with cell toxicity [15]. Although not necessary to incorporate high amounts of RT in FV particles, this abundance of FV Pol proteins in infected cells may have other yet undiscovered reasons in FV biology.

### Determination of the Pol protein amounts relative to Gag in FV particles

We generated highly purified virus by consecutive centrifugation through a sucrose cushion and a linear gradient made of iodixanol. BHK-21 cells were infected with the supernatant from transfected 293T cells and cell-free virus was harvested when productive infection was ongoing, usually after 3–5 days. The supernatant was clarified from cellular debris by low-speed centrifugation and filtered through a 0.45μm pore-size filter (Sartorius). Virus was concentrated by centrifugation through a 20% sucrose cushion in TNE buffer (20 mM TRIS-HC1, pH 7.5, 150 mM NaC1, 1 mM EDTA) in a SW28 rotor (Beckman) at 25,000 rpm, 4°C for 1 hr. The sediment was resolved in Dulbecco's minimal essential medium (DMEM) and placed on a 2 ml 10–40% continuous iodixanol (OptiPrep from Axis-Shield) gradient for further virus purification. The gradient was cast in a gradient mixer (SG30 from Hoefer) the day before use. Following centrifugation in a TLS-55 rotor (Beckman) at 48,000 rpm and 4°C for 4 hrs, 200 μl fractions were taken from the top. From each fraction 30 μl were used for the determination of the refraction index, 20 μ1 for infectivity assay on BHK/LTR(PFV)lacZ cells [16], and l00 μl for immunoblotting.

As exemplified in Fig. 3A, fractions 5 and 6 were the main gradient fractions in which viral Gag and Pol proteins were detected by immunoblotting. Fraction 6 was also the main fraction of viral infectivity as shown in Fig. 3B. A mean density of 1.119 g/ml (± 0.011) was found for infectious PFV particles. This value is slightly lower than previous results with sucrose gradients [3,17,18]. Defined amounts of bacterially-expressed Gag and Pol proteins were also applied to the gel. The intensities of the bands were determined with a LAS-3000 (Fujifilm) and the relative amounts of Gag and Pol proteins were calculated using the software Image Gauge 3.01 (Fujifilm). A regression curve was formed, in which the total amounts of recombinant protein loaded in each lane were related to the optical densities of the individual protein bands which were produced after blotting, reaction with MABs, and ECLplus staining. In Fig. 4 an example is depicted, which was derived from the same samples shown in Fig. 3. The ability to build a regression curve from the sample detection also illustrates that the assay was linear over the protein range analyzed.

**Figure 3:**
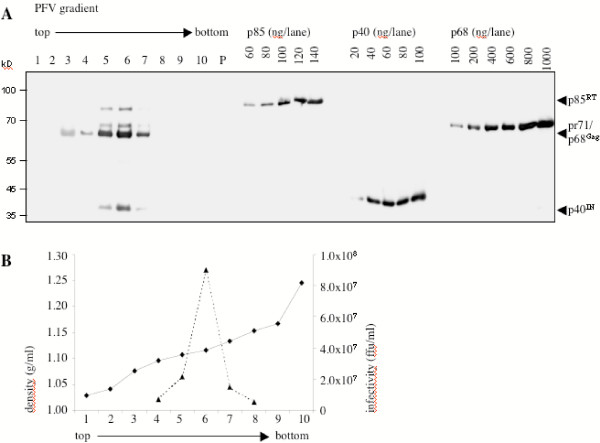
Representative example of the determination of the relative amounts of Gag and Pol proteins in purified PFV. (A) Extracellular virus was centrifuged through a sucrose cushion and the sediment was loaded onto a linear iodixanol gradient. Fractions were taken from the top and analyzed by immunoblotting with the Gag- and Pol-specific MABs. Defined amounts of recombinant PFV Gag and Pol proteins were also loaded onto the gel and simultaneously incubated with the MAB solutions. The blot was developed with the ECLplus reagent from Amersham-Pharmacia. (P), Pellet of the gradient. (B) Density and infectivity of the gradient fractions shown in (A). The infectivity was determined by a blue cell assay [16].

**Figure 4:**
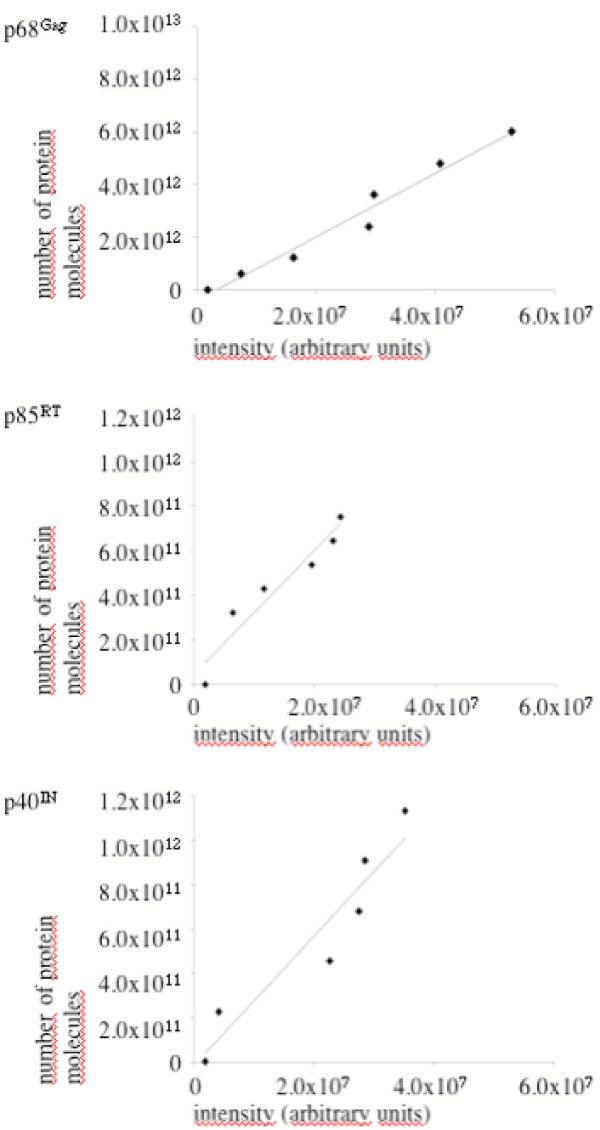
Relation of the intensities of the bands in the lanes with recombinant PFV proteins shown in Fig. 3 and amounts of protein loaded onto the gel. The latter was expressed as the number of molecules. Band intensities were determined with a LAS-3000 and calculated using the Image Gauge 3.01 software (Fujifilm). Over the protein range analyzed the band intensities were found to be in a linear relation to the protein amounts.

A total of 36 gradient fractions were analyzed with three independent quantifications for the individual gradients. The results are summarized in Table 1. We found that purified FV virions had a mean pr71^Gag ^to p68^Gag ^ratio of 1 to 4.2, which indicated that the cleaved p68^Gag ^protein is the dominant capsid protein species in infectious PFV particles. The SGG1 MAB binding site is located N-terminal of the Gag cleavage site that generates p68 Gag and the 3 kD C-terminal peptide from the pr71 Gag precursor (our unpublished results). Therefore, the antibody detects both, the uncleaved and the cleaved protein equally well. The 127 kD Pol precursor protein was barely detected in the virus preparations, which indicated almost complete cleavage into the 85 kD RT and 40 kD IN subunits. Importantly, the relation of Gag proteins (pr71 plus p68) to p85^RT ^was determined to be 15.8 to 1. This illustrates that PFV has found an independent way to incorporate as much Pol protein relative to Gag into progeny virus as typically found in orthoretroviruses [19]. With respect to the amount of IN protein, a ratio of 9.8 Gag molecules (pr71^Gag ^plus p68^Gag^) to 1 IN molecule was revealed. Considering only the cleaved moiety, the p68^Gag^/p40^IN ^ratio was determined to be 7.8 to 1 (Table 1). Thus, we constantly detected approximately 1.6 to two times more IN than RT protein in infectious virions. FV initially encapsidate the 127 kD Pol precursor protein which is cleaved into its subunits after packaging [4]. It may, therefore, be surprising not to find equal amounts of the two subunits in virions. The reason for this is presently unclear. It may be that different blotting efficiencies of the two proteins account for differences in detectability. Alternatively, different amounts of RT and IN enzymes in viral particles may be a consequence of the particular FV replication pathway. FVs reverse transcription takes place to a significant extent in the cytoplasm before progeny virus release [12,20,21]. The conditions of this reverse transcription late in the replication cycle are not understood. *Gag *gene expression appears to be required [22,23], but complete assembly of viral capsids may be not. While IN enzyme will be needed by the virus for the next round of replication, the RT subunit may be dispensable to the extent reverse transcription has already been completed and there is no need for RT to be actively encapsidated.

**Table 1:** Relative amounts of Gag and Pol proteins in foamy viruses

	pr71/p68^Gag^:p85^RT^	p68^Gag^:p85^RT^	Pr71/p68^Gag^:p40^IN^	p68^Gag^:p40^IN^	p68^Gag^:pr71^Gag^
**Mean**	15.8 : 1	12.3 : 1	9.8 : 1	7.8 : 1	4.2 : 1
**SD^1^**	5.6	4.8	7.8	6.9	2.0
**Maximum**	26.3 : 1	22.7 : 1	41.3 : 1	35.8 : 1	8.0 : 1
Minimum	6.8 : 1	5.2 : 1	3.0 : 1	2.3 : 1	1.3 : 1

^1^SD, standard deviation

As detailed above, the reasons to assume that only trace amounts of Pol protein are encased in spumaretrovirus virions were hitherto largely theoretical. We provide here experimental evidence that many more Pol molecules per capsid can be found in purified FVs than was previously thought, even when taking into account that we did not determine the absolute numbers of molecules per virion, but only the relative Gag to Pol ratios. How can this finding be explained in the light of recent results in which two distinct RNA structures were identified to be essential for Pol protein incorporation into FV particles [4]? Firstly, with respect to this study only the minimal RNA sequence requirements for Pol protein encapsidation using subgenomic constructs have been determined, and not the relative ratios between Gag and Pol using a full-length viral genome. Secondly, it may be that the presence of the RNA domains, found to be responsible for Pol packaging, leads to the encapsidation of not only two Pol molecules per viral RNA, but of a larger complex which consists of many more protein molecules. This complex may be stabilized by protein-protein interactions between Pol and Gag, the individual Pol molecules, or a combination of both.

## Authors' contributions

MC performed all experiments described in this manuscript. WR assisted in bacterial expression and purification of recombinant proteins. The experiments were designed and supervised by OH, DL and AR. AR wrote the manuscript together with MC.
